# 5 years after the Kahn's etiquette-based medicine: a brief checklist proposal for a functional second meeting with the patient

**DOI:** 10.3389/fpsyg.2013.00723

**Published:** 2013-10-23

**Authors:** Gianluca Castelnuovo

**Affiliations:** ^1^Psychology Research Laboratory, Istituto Auxologico Italiano IRCCS, Ospedale San GiuseppeVerbania, Italy; ^2^Department of Psychology, Catholic University of MilanMilan, Italy

**Keywords:** etiquette-based medicine, communication, clinical psychology, relationships, strategy

5 years ago, in a widespread magistral editorial on NEJM (Kahn, 2008), Kahn discussed that medical and clinical education and postgraduate training have to better take into account the so called “etiquette-based medicine.” Kahn provided an interesting and practical checklist for physicians and health professionals to improve “etiquette” in the first meeting with a patient:

1. Ask permission to enter the room; wait for an answer.
2. Introduce yourself, showing ID badge.
3. Shake hands (wear glove if needed).
4. Sit down. Smile if appropriate.
5. Briefly explain your role on the team.
6. Ask the patient how he or she is feeling about being in the hospital.” (p 1988, Kahn, 2008).

The main goal of this commentary is to provide a brief checklist proposal for a possible second meeting with the patient, particularly when prescriptions and therapeutic indications provided in the first meeting were not totally or partially followed.

In the second meeting please ask yourself the following question: “Where was my behavior lacking in the first meeting? In the “relationship,” “communication,” or “strategy” level?”

1. If I was lacking in the relationship, perhaps it means the my patient does not rely on me and I have to recover or rebuild a good relationship showing empathy, professionalism and giving attention to the entire person (the biopsychosocial dimension of each human being) more than to the single pathology problem, allowing patients to continue expressing emotions (Pollak et al., 2007).
2. If I was lacking in the communication, perhaps it means the I have not well communicated my indications. It could be that I have not used the client's language: for some patients rational and demonstrative communications could be useful, whereas for other ones evocative and persuasive elements are necessary (Bailey, 2006; Pagnini et al., 2009; Castelnuovo, 2013).
3. If I was lacking in the strategy, perhaps it is also necessary to take into account the patient's type of resistance to change. It is crucial to distinguish among collaborative patients, non-collaborative ones and openly opposing ones: for each category different strategies are required [please find more suggestions in (Bailey, 2006)].

So psychology can deepen and train the relationship, communication, and strategy skills necessary to improve the clinical procedures and results not only in a first etiquette-based meeting, but also during the following etiquette-based treatment sessions (Castelnuovo, 2010).
